# Regulation of Immune Responsiveness *In Vivo* by Disrupting an Early T-Cell Signaling Event Using a Cell-Permeable Peptide

**DOI:** 10.1371/journal.pone.0063645

**Published:** 2013-05-07

**Authors:** David M. Guimond, Nicholas R. Cam, Nupura Hirve, Wei Duan, John D. Lambris, Michael Croft, Constantine D. Tsoukas

**Affiliations:** 1 Department of Biology and Center for Microbial Sciences, San Diego State University, San Diego, California, United States of America; 2 Division of Immune Regulation, La Jolla Institute for Allergy and Immunology, San Diego, California, United States of America; 3 Department of Pathology and Laboratory Medicine, University of Pennsylvania School of Medicine, Philadelphia, Pennsylvania, United States of America; French National Centre for Scientific Research, France

## Abstract

The inducible T cell kinase (ITK) regulates type 2 (Th2) cytokines that provide defense against certain parasitic and bacterial infections and are involved in the pathogenesis of lung inflammation such as allergic asthma. Activation of ITK requires the interaction of its SH3 domain with the poly-proline region of its signaling partner, the SH2 domain containing leukocyte phosphoprotein of 76 kilodaltons (SLP-76). The specific disruption of the ITK-SH3/SLP-76 poly-proline interaction *in vitro* by a cell-permeable competitive inhibitor peptide (R9-QQP) interferes with the activation of ITK and the transduction of its cellular functions in T lymphocytes. In the present investigation, we assessed the effects of R9-QQP treatment on the induction of an in vivo immune response as represented by lung inflammation in a murine model of allergic asthma. We found that mice treated with R9-QQP and sensitized and challenged with the surrogate allergen ovalbumin (OVA) display significant inhibition of lung inflammation in a peptide-specific manner. Thus, parameters of the allergic response, such as airway hyper-responsiveness, suppression of inflammatory cell infiltration, reduction of bronchial mucus accumulation, and production of relevant cytokines from draining lymph nodes were significantly suppressed. These findings represent the first demonstration of the biological significance of the interaction between ITK and SLP-76 in the induction of an immune response in a whole animal model and specifically underscore the significance of the ITK-SH3 domain interaction with the poly-proline region of SLP-76 in the development of an inflammatory response. Furthermore, the experimental approach of intracellular peptide-mediated inhibition might be applicable to the study of other important intracellular interactions thus providing a paradigm for dissecting signal transduction pathways.

## Introduction

The Inducible T cell Kinase (ITK) is a member of the Tec family of tyrosine kinases [1] that is upregulated during type 2 helper T cell (Th2) differentiation [2], [3]. Although ITK does not appear to be essential for the development of Th2 cells per se, it is critical for the expression of Th2 cell effector function, as evidenced by defective expression of Th2 cytokines and the transcription factor GATA3 in ITK deficient animals [4]. In view of its role in Th2 cytokine regulation, ITK has been also implicated in the defense against certain parasitic and bacterial infections and in the pathogenesis of lung inflammation such as allergic asthma [5]–[8].

For its enzymatic activation, ITK requires interaction with the adaptor protein SLP-76 that is induced by the engagement of the T Cell Receptor (TCR) for antigen [9]. In the absence of this interaction, ITK does not become recruited to the appropriate intracellular sites and, consequently, it does not become trans-phosphorylated and enzymatically activated [9]–[12]. The association between ITK and SLP-76 is co-operative and involves the interaction between the ITK-SH2 domain and pTyr145 on SLP-76, as well as the interaction between the ITK-SH3 domain and its polyproline ligand on SLP-76 [9], [10], [13]. In recently published *in vitro*/*ex vivo* studies from our laboratory, we disrupted the interaction of ITK and SLP-76 using a novel cell-permeable peptide (denoted R9-QQP) representing the minimal binding motif of the polyproline domain of SLP-76 that interacts with the SH3 domain of ITK [12]. Upon TCR-mediated activation of T cells that had been treated with R9-QQP, ITK failed to become recruited to the appropriate intracellular site, its enzymatic activity was reduced, and its ability to transduce the production of Th2 cytokines was compromised [12]. Importantly, these effects were peptide-specific, as separate control peptides displayed no statistically significant effects [12]. Furthermore, the effects of R9-QQP were selective for the ITK/SLP-76 interaction because biochemical interactions between SLP-76 and other SH3 domain-containing proteins known to interact with the poly-proline region of SLP-76 were not affected [12].

The significance of the interaction between ITK and SLP-76 in an in vivo induced immune response has not been previously assessed. In the present investigation, we have investigated the effects of R9-QQP treatment on an immune response mediated by Th2 cytokines, namely lung inflammation as manifested in a murine model of allergic asthma. We found that mice treated with R9-QQP and sensitized and challenged with the surrogate allergen ovalbumin (OVA) display significant inhibition of lung inflammation in a peptide-specific manner. Parameters of the allergic response, such as airway hyper-responsiveness, suppression of inflammatory cell infiltration, reduction of bronchial mucus accumulation and production of relevant cytokines from draining lymph nodes were significantly suppressed.

The data presented here provide the first *in vivo* evidence for the biological significance of the interaction between ITK and SLP-76 and underscore the importance of the interaction between the SH3 domain of ITK and the poly-proline region of SLP-76 in the generation of an immune response in a whole animal model. The experimental approach using cell-permeable peptides as competitive inhibitors might be also applicable to the study of other important intracellular interactions thus providing a paradigm for dissecting intracellular signal transduction.

## Materials and Methods

### Ethics Statement

All protocols using mice were carried out in strict accordance with the recommendations in the Guide for the Care and Use of Laboratory Animals of the National Institutes of Health. Protocols were approved by the Institutional Animal Care and Use Committees of San Diego State University (A3728-01) and La Jolla Institute for Allergy and Immunology (A3779-01).

### Animals

**Figure 1:**
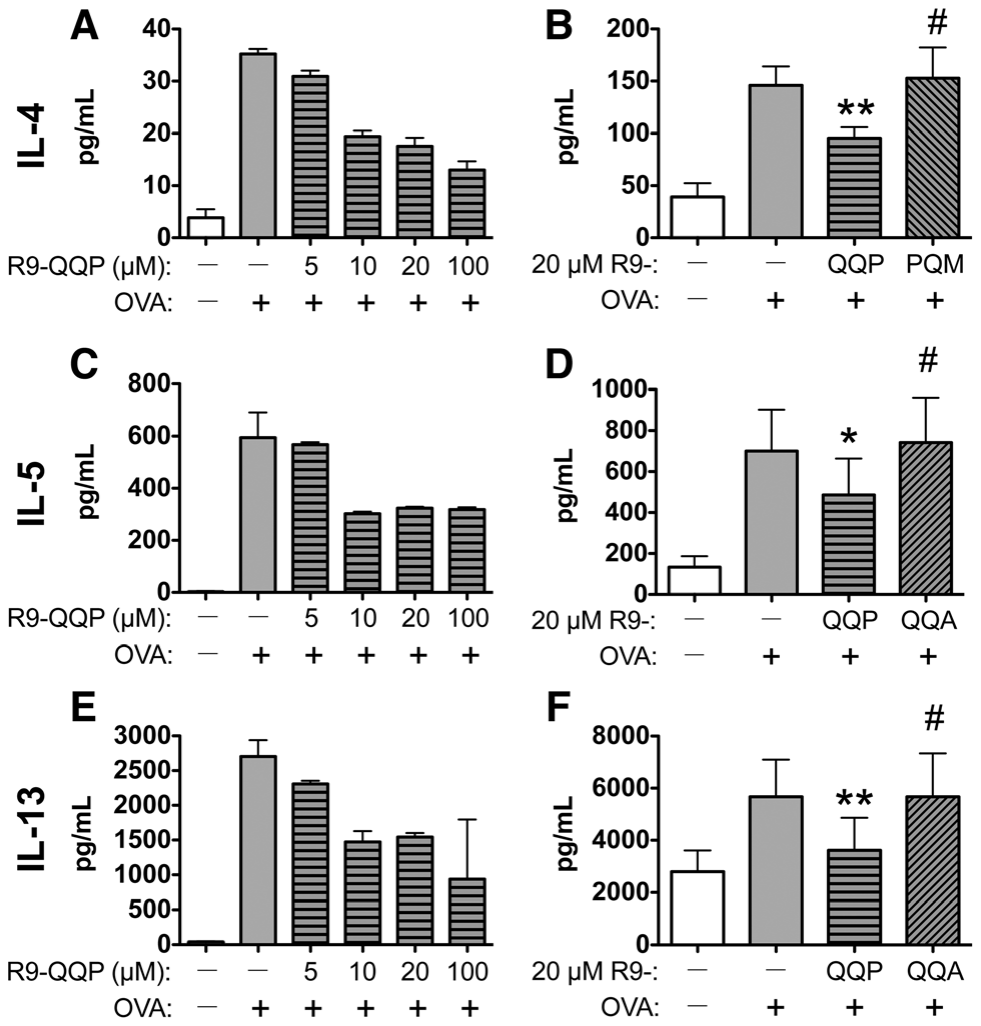
R9-QQP inhibits OVA-inducible Th2 cytokine production in a dose-dependent and peptide-specific manner. Splenocytes, obtained from mice seven days following i.p. immunization with 20 µg OVA + 2 mg alum, were treated with various concentrations of R9-QQP or without peptide and re-stimulated *in vitro* with 50 µg/mL OVA as indicated. After incubation for 4 days, cell-free tissue culture supernatant samples were assayed for IL-4 (A), IL-5 (C) and IL-13 (E) by ELISA as described in Materials and Methods. Results are those from single titrations for each cytokine and are displayed as the average (± S.D.) of duplicate determinations. For assessing peptide specificity, similar cultures were established as above and splenocytes were treated with 20 µM of R9-QQP, R9-PQM, R9-QQA or no peptide as indicated, and culture supernatants assayed for IL-4 (B), IL-5 (D) and IL-13 (F). Results are displayed as the average (± S.E.M.) of 5-6 replicate experiments utilizing different mice. For each cytokine tested, statistical evaluations were determined by a paired student's t test comparing peptide-treated cells to vehicle-treated/OVA-restimulated cells. *, p < 0.05; **, p < 0.01; #, p>0.05.

Female BALB/c (experiments presented in Figure 1) or C57BL/6 (all other experiments) mice between the ages of 6-12 weeks were purchased from Jackson Laboratories (Bar Harbor, ME) and were housed in microisolator cages until used for experiments.

### Cell-permeable synthetic peptides

RRRRRRRRRQQPPVPPQRPMA (R9-QQP), RRRRRRRRRPQMPAPQRPQPV (R9-PQM) and RRRRRRRRRQQAAVAAQRAMA (R9-QQA) peptides were synthesized by GL Biochem, Ltd. (Shanghai, China) using L-enantiomer amino acids as we have previously described [12]. Peptides were purified to >95% purity and their mass was confirmed by matrix-assisted laser desorption mass spectrometry. Peptides were reconstituted in cell culture grade PBS (Mediatech Cellgro/Corning, Inc.; Manassas, VA) for in vivo administration or in cell culture grade water (HyClone/Thermo Fisher Scientific, Inc.; South Logan, UT) for in vitro cultures. R9-PQM is a scrambled sequence of QQP amino acids whereas R9-QQA represents substitution of all Pro with Ala. Both control peptides behaved similarly and have been used interchangeably in the experiments presented here.

### Sensitization to OVA, asthma induction, and peptide treatment

**Figure 2:**
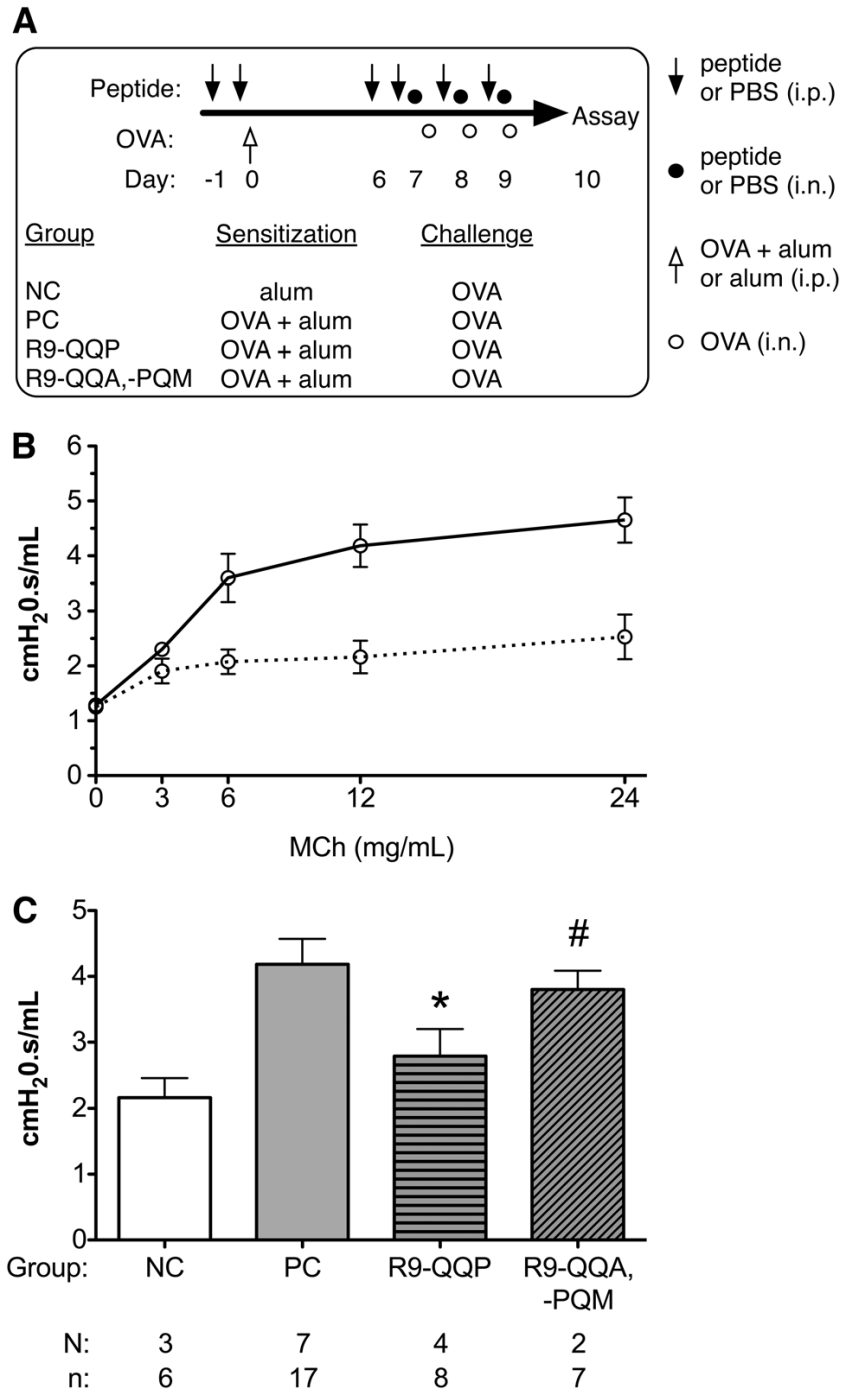
Mice treated with R9-QQP display reduced airway hyperresponsiveness (AHR). (A) Regimen of OVA immunization and challenge, and peptide treatment with description of treatment for each group of mice as detailed in Materials and Methods. Abbreviations used to represent control groups of mice used for this and subsequent figures are defined as follows: NC = negative control, PC = positive control. (B) Twenty-four hours after the final challenge with OVA, airway resistance was measured for each mouse using the Scireq FlexiVent system in response to increasing concentrations of aerosolized methacholine (MCh). Results represent the group average (± SEM of independent experiments) airway resistance values representing 6 non-sensitized (^……^) and 17 OVA-sensitized (^____^) mice in response to various concentrations of MCh. (C) Airway resistance was measured as in panel B using groups of mice that had been rendered asthmatic and treated with R9-QQP or the control peptides R9-QQA or R9-PQM (data were pooled for the two control peptides) or with vehicle as defined in panel A. Bar graphs display the average (± SEM of independent experiments) airway resistance in response to 12 mg/mL MCh. ‘N’ represents the number of independent experiments and ‘n’ represents the total number of animals for each group. *, p<0.05; #, p>0.05.

Sensitization to and challenge with the model allergen ovalbumin (OVA, grade V; Sigma; St. Louis, MO; Cat. # A-5503) was performed by intraperitoneal (i.p.) injection of C57BL/6 mice with 20 µg OVA/2 mg alum (Pierce/Thermo Scientific, Inc; Rockford, IL; Cat. # 77161). In order to induce asthma, mice sensitized to OVA were subsequently challenged by intranasal (i.n.) instillation of 10 µg OVA/20 µL PBS into anesthetized mice on days 7, 8, and 9 post sensitization. For peptide treatment, mice sensitized and challenged with OVA as above were treated with R9-QQP or control peptides (R9-PQM or R9-QQA) by i.p. injection of 25 mg peptide per kg of mouse weight (∼0.5 mg/mouse) 24 hours and again 30 minutes before OVA sensitization. This was followed by additional i.p. injections of peptides 24 hours before each intranasal challenge with OVA on days 7, 8, and 9 and by i.n. delivery (200 µg in 20 µl PBS) 2 hours before intranasal OVA challenge. According to the above protocol total peptide delivery was 2.5 mg i.p. and 0.6 mg i.n. per mouse. On day ten of the protocol mice and collected tissues were used for assessment of various asthma parameters. The regimen of OVA sensitization and challenge, and treatment with peptide is outlined in Figure 2A.

### Restimulation of OVA sensitized lymphoid cells *in vitro*

Lymphoid cells were isolated from the spleens of mice that had been sensitized to OVA, as described above, but not treated with peptide. The spleens were mechanically dissociated and red blood cells removed by incubation in lysis buffer (0.15 M NH_4_Cl, 10 mM KHCO_3_, 0.1 mM Na_2_EDTA) for 5–10 minute at room temperature. After washing, the cells were treated with various concentrations of R9-QQP or control peptides for 30 minutes at 37°C as previously described [12]. Following peptide treatment, 1×10^6^ cells were cultured in round-bottom 96-well plates (Sarstedt, Cat. # 83.1837; Nümbrecht, Germany) along with 50–100 µg/ml OVA in total volume of 200 µl of RPMI, 10 mM HEPES, 2 mM L-glutamine, 100 I.U./mL penicillin, 100 µg/mL streptomycin and 50 µM 2-mercaptoethanol (2-ME; Sigma) for 4 days at 37°C in a 5% CO_2_ humidified atmosphere. Cell free supernatants were assessed for the presence of cytokines as described below. In other experiments, lymphoid cells were isolated from the peribronchial and mediastinal lung-draining lymph nodes (DLN) of mice that had been sensitized and challenged with OVA and treated with the peptide regimen described above. Cells, 10^5^–10^6^ per microtiter tray well, were restimulated with OVA, as described above for splenocytes, but without any in vitro peptide treatment. Culture supernatants were assessed for cytokine production.

### Assessment of cytokines

IL-4, IL-5, and IL-13 in culture supernatants from in vitro OVA-restimulated lymphoid cells were quantified by sandwich ELISA utilizing commercially obtained kits and following the manufacturer's recommendations (eBioscience, Inc. San Diego, CA). All assays had a detection limit of 4 pg/ml and analysis was performed in duplicate in microtiter well plates (BD Biosciences, Cat. # 353279) following the manufacturer's recommendations (eBioscience, Inc.). Plates were analyzed using the eMax Precision Microplate Reader and SoftMax Pro v5.0 software (Molecular Devices, Sunnyvale, CA).

### Measurement of airway hyperresponsiveness (AHR)

Asthma induction was performed as described above and invasive airway hyperresponsiveness (AHR) of peptide- or control-treated mice was measured 24 hours after the final challenge with OVA using the FlexiVent small rodent ventilator system (Scireq, Inc.; Montreal, QC, Canada). Calibration of the FlexiVent system was performed prior to acquiring data for each subject according to the manufacturer's instructions. First, mice were anesthetized by i.p. injection of a cocktail containing 80 mg/kg of ketamine (∼2 mg; Fort Dodge Animal Health; Overland Park, KS) and 8 mg/kg of xylazine (∼0.2 mg; Lloyd, Inc.; Shenandoah, IA). Next, the trachea was exposed and a small incision was created at the location of the cartilage ring; tracheotomy was then performed by insertion of an 18 gauge cannula (BD Medical, Cat. # 408208; Franklin Lakes, NJ) that was secured using surgical suture. Following intratracheal cannulation, the adaptor end of the cannula was connected to the FlexiVent system and ventilation was initiated. Once mice were passively breathing, airway resistance was measured in response to increasing doses of aerosolized methacholine (Sigma, Cat. # A2251) that were sequentially delivered to the lungs through the endotracheal cannula. The maximal airway resistance values for each dose of methacholine were recorded for each mouse and averaged (± SEM) for all mice belonging to the same treatment group for each independent experiment.

### Collection of BAL fluid and lung tissue dissociation

Mice were treated following the asthma protocol described above and bronchoalveolar lavage fluid (BALF) was collected 24 hours after the final challenge with OVA. For collection of BALF, tracheotomy was performed as described above and an endotracheal cannula was inserted to a depth of 1 cm beyond the point of incision. One mL of ice-cold PBS was then slowly dispensed to the lungs and aspirated, repeating the process five times. Next, cells were collected by centrifugation and RBCs were lysed using lysis buffer as described above. After performing lavage, lung lobes were excised, rinsed in PBS and minced into 1–2 mm pieces that were digested for 1 hr at 37°C in tissue culture medium containing 3 mg/mL collagenase (Worthington Biochemical Corp., Cat. # LS004186; Lakewood, NJ) and 100 µg/ml DNAase I (Sigma, Cat. # DN25). The tissue was then mechanically dissociated and the cell suspension was collected and washed in 0.5% BSA/PBS + 0.02% NaN_3_.

### Characterization of cellular infiltrates

BAL cells collected as described above were characterized by preparing cytospin slides using a Shandon Cytospin 2 centrifuge (Thermo Scientific, Inc.; Waltham, MA) and were stained with a modified Wright-Giemsa dye according to the manufacturer's instructions (Protocol/Fisher HealthCare; Houston, TX; Cat., # 122-911). Alveolar macrophages were identified by light microscopy as large cells with atopic nuclei and cytoplasm staining purple while eosinophils were identified as small cells with bi-lobed nuclei and cytoplasmic granules staining pink.

In addition, cellular infiltrates were quantified for viable cells by erythrosine B (0.15%) staining and counting in a hemocytometer and percentage of subpopulations were assessed by staining with specific fluorochrome-conjugated antibodies (BD Biosciences, San Jose, CA) and analyzed by flow cytometry. Prior to staining, cell surface Fc receptors were blocked by incubation of samples with 0.5 µg of rat anti-mouse CD16/CD32 (Cat. # 553141). Staining for eosinophils and macrophages was performed according to the method described by Stevens et al [14] using a cocktail containing 0.5 µg each of PE-conjugated rat anti-Siglec-F (Cat. # 552126), PerCP-Cy5.5-conjugated rat anti-CD45 (Cat. # 550994) and FITC-conjugated hamster anti-mouse CD11c (Cat. # 557400). Fluorescence of stained cells was acquired using a FACSCanto flow cytometer (BD Biosciences) and FACS analysis was performed using FlowJo software (Tree Star, Inc., Ashland, OR) as follows: first, samples were gated for CD45^+^ cells that were subsequently analyzed for expression of Siglec-F and CD11c. Eosinophils and macrophages were both positively defined by expression of Siglec-F and were differentially gated based on low versus high expression of CD11c, respectively. Results are expressed as eosinophil and macrophage percentages of CD45^+^ cells.

Similarly, BAL cells were stained for quantification of T lymphocytes using a cocktail containing 0.5 µg each of PerCP-Cy5.5-conjugated rat anti-CD45 and PE-Cy7-conjugated hamster anti-CD3ε antibodies (Cat. # 552774). Fluorescence of stained cells was acquired using a FACSAria flow cytometer (BD Biosciences) and FACS analysis was performed as follows: first, cells were gated for CD45^+^ events followed by further defining lymphocytes based on their characteristic scatter profile (FSC/SSC). Lastly, CD45^+^ cells within this scatter profile were further analyzed for TCR/CD3 expression and T lymphocytes were expressed as a percentage of total CD45^+^ cells.

For quantification of the absolute number of eosinophils, macrophages and T cells, the percentage of each sub-population as determined by FACS analysis was multiplied by the total number of BAL cells as determined by hemocytometer counts obtained for each mouse.

### Histological examination

Separate lung lobes representing each treatment group were fixed in 10% zinc formalin (Protocol/Fisher HealthCare, Cat. # 313-095) and 5 µm paraffin sections were prepared and analyzed for the presence of mucus by staining with periodic acid Schiff (PAS) reagent and counterstaining with hematoxylin. Sample preparation was performed by Pacific Pathology (San Diego, CA). Goblet cell hyperplasia and mucus production were evaluated by assigning a mucus severity score according to the percentage of each bronchiole cross section staining fuchsia as follows: a value of 0 indicates no detectable mucus, a value of 1 indicates≤10% staining positive for mucus, a value of 2 indicates>10% and≤25% staining positive, a value of 3 indicates>25% and≤50% staining positive, a value of 4 indicates>50% and≤75% staining positive and a value of 5 indicates>75% and≤100% staining positive.

Recruitment of inflammatory cells to the lungs was visualized by staining slides prepared as described above with hematoxylin and eosin (H&E). The degree of inflammation was evaluated by assigning a score according to the depth of infiltrating cells surrounding each blood vessel or bronchiole as follows: a value of 0 indicates no infiltrating cells, a value of 1 indicates a single layer of cells, a value of 2 indicates a layer of 2–4 cells, a value of 3 indicates a layer of 5–7 cells and a value of 4 indicates a layer of ≥ 7 cells.

All of the above evaluations were performed blindly by randomly coding slides prior to visualization by light microscopy using a 40×objective.

### Data Analysis

Statistical analysis (student's t test) and graphical representation of data was performed using Prism software version 5.0 (GraphPad Software; La Jolla, CA). Statistical evaluation of all asthma parameters were determined by a student's t test comparing each peptide-treated group to the positive control (PC) group; a p value < 0.05 was considered statistically significant. For lung histological analyses, raw mucus severity and inflammation scores obtained for each peptide treatment group were normalized to the average PBS/vehicle control treatment, respectively, and values for each group were graphically represented as percentage of control.

## Results

### Treatment with R9-QQP inhibits OVA-dependent cytokine production by effector T cells

In our previously published *in vitro* studies in which we found that R9-QQP was able to inhibit ITK activation and compromise Th2 cytokine production, we activated T cells with an anti-TCR stimulatory antibody [12]. Prior to applying the R9-QQP inhibitory peptide in the mouse model of allergic asthma, it was important to demonstrate the ability of R9-QQP to inhibit a T-cell response to an antigen (OVA) that would be later used as a surrogate allergen. To this end, splenocytes isolated from mice that had been sensitized to OVA were treated with R9-QQP and restimulated with OVA *in vitro* in order to assess cytokine production as part of a T cell-dependent recall response to antigen. Treatment with R9-QQP resulted in a peptide dose-dependent inhibition of three Th2 signature cytokines (IL-4, -5 and -13) that have been implicated in lung inflammation [15] with optimal inhibitory concentration greater than 5 µM (Figures 1A, C, and E). Importantly, when 20 µM of either control peptide (R9-PQM or R9-QQA) was compared to the same amount of R9-QQP, there was no inhibition (Figures 1B, D, F). It should be noted that the variation in cytokine levels seen in the titration experiments (Figures 1A, C, and E), where C57BL/6 mice were used, and the peptide specificity experiments (Figures 1B, D, and F) where Balb/c mice were used, is consistent with the propensity for higher levels of Th2 cytokine production seen with Balb/c mice. Under our experimental conditions we could not detect an OVA-inducible IFN_γ_ production, therefore the effect of R9-QQP on this Th1 cytokine could not be assessed. These data indicate that the inhibitory effect of R9-QQP is peptide cargo sequence-specific and also that the inhibition was directed on effector T-cell function since the *in vitro* stimulated cells were obtained from animals that had been previously exposed to OVA *in vivo*. Furthermore, the lack of inhibition seen with the control peptide cannot be attributed to an inability of peptide to enter cells as studies with FITC-labeled peptides have shown that R9-QQP as well as both R9-PQM and R9-QQA control peptides can be internalized (ref. [12] and data not shown).

### Inhibition of ITK by R9-QQP reduces lung inflammation-induced airway hyper-responsiveness (AHR)

Lung inflammation, Th2 cytokine production, goblet cell hyperactivity and mucus production are distinctive features of airway allergic conditions such as bronchial asthma [16]. In terms of lung function, these events contribute to the pathophysiological condition of airway hyper-responsiveness [16]. We wished to determine whether inhibition of ITK activation by R9-QQP interferes with this pathophysiological parameter. To this end, we utilized an invasive AHR measurement system (FlexiVent small rodent ventilator system, Scireq Inc.) in which airway resistance is measured in response to increasing doses of aerosolized β-methacholine administered to anesthetized mice by endotracheal delivery. First, we established a methacholine dose response in order to determine an optimal amount of the drug. The data shown in figure 2B established 12 mg/ml as a dose of methacholine on the plateau of the dose-response curve. Under these conditions, OVA-allergic mice treated with R9-QQP according to the regimen outlined in Figure 2A and detailed in Materials and Methods displayed a significant decrease in AHR (Figure 2, compare R9-QQP and PC bars; p<0.05). Treatment with R9-QQP decreased AHR to levels close to those of mice that were not sensitized (Figure 2C, compare R9-QQP and NC bars; p = 0.2952). Importantly, such a decrease was not seen when mice were treated with the control peptides (Figure 2C).

### R9-QQP reduces mucus production and lung inflammation

**Figure 3:**
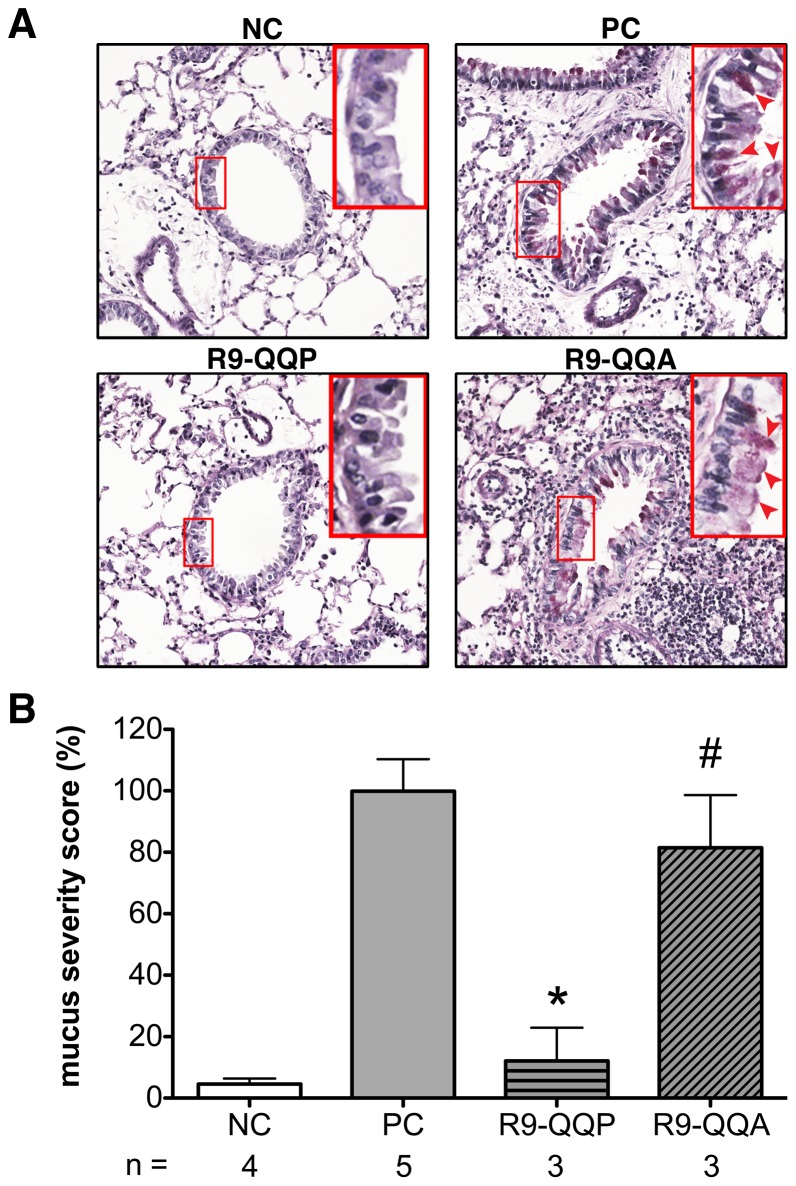
R9-QQP specifically inhibits mucus production by goblet cells surrounding bronchioles. (A) Representative micrographs (200×magnification) of bronchioles prepared by staining (PAS) cross-sections of lung tissue of mice that had been treated as indicated in Figure 2A and detailed in Materials and Methods. Inset boxes display additional digital magnification of corresponding region outlined in original image and red arrowheads indicate the presence of mucus producing goblet cells as defined by cytoplasm staining fuchsia. (B) Normalized averages (± SEM) of mucus severity scores for the indicated number (n) of mice calculated as described in Materials and Methods; for each mouse, approximately 30 bronchioles were stained and analyzed as in panel A. 100% mucus severity score correlated with a raw score of 3.3, assessed as described in Materials and Methods. *, p < 0.05; #, p>0.05.

As mentioned above, the asthmatic response is characterized by mucus accumulation as well as the recruitment of inflammatory infiltrates to the lungs [16]. In the current study, we observed that mucus production was reduced in the bronchi of OVA-allergic mice that were treated with R9-QQP (Figure 3A; compare PC and R9-QQP panels) while mice treated with the control peptide R9-QQA were unaffected (Figure 3A; compare PC and R9-QQA panels). As displayed in Figure 3B, cumulative data from multiple experiments demonstrated that treatment with R9-QQP reduced the mucus severity score by 88% as compared to OVA-allergic mice that were treated with vehicle (PC). In addition, treatment with control peptide R9-QQA did not result in a significant reduction of the mucus severity score for this group of mice.

**Figure 4:**
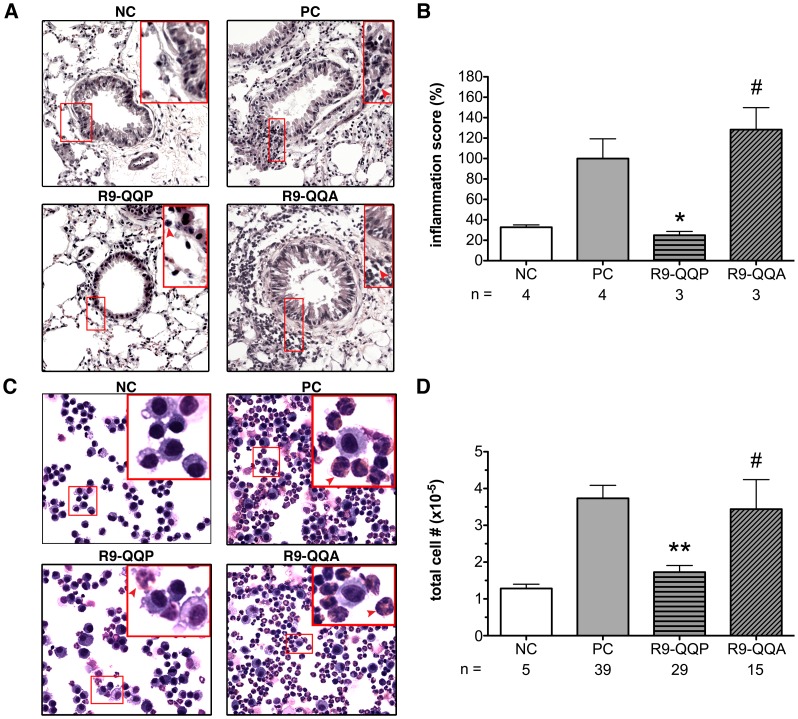
Mice treated with R9-QQP display reduced lung inflammation. (A) Representative micrographs (200×magnification) of bronchioles prepared by staining (Hematoxylin and Eosin) cross sections of lung tissue of mice that had been treated as indicated in Figure 2A and detailed in Materials and Methods. Inset box in upper right corner of each micrograph displays additional digital magnification of corresponding region outlined in original image. Peribronchial inflammatory cells are identified by dark purple staining of nuclei and are indicated by red arrowheads. (B) Normalized averages (± SEM) of inflammation scores for the indicated number (n) of mice calculated as described in Materials and Methods; for each mouse, approximately 45 bronchioles and blood vessels were stained and analyzed as in (A). 100% inflammation score correlated with a raw score of 2.3, assessed as described in Materials and Methods. (C) Representative micrographs (400×magnification) of cells obtained from the BAL fluids of mice treated as described in Figure 3 and stained with Wright-Giemsa dye. Inset boxes in upper right corner of each micrograph shows additional digital magnification of corresponding region outlined in original images. Arrowheads indicate eosinophils as identified by the presence of red cytoplasmic granules. (D) Average (± SEM) of total cell numbers obtained from the indicated (n) number of mice under each experimental condition. *, p < 0.05; **, p < 0.0001; #, p>0.05.

Goblet cell hyperactivity and mucus accumulation are triggered by the infiltration and accumulation of inflammatory cells that release soluble lipid mediators and sustained production of pathogenic Th2 cytokines [17]. Treatment of OVA-allergic mice with R9-QQP resulted in 71% reduction in the inflammation score as assessed in lung tissue sections that were stained with hematoxylin and eosin (Figure 4A and B). This observation was consistent with the reduction in total cell numbers seen in BAL fluids (Figure 4C and D). Furthermore, microscopic evaluation of inflammatory cells present in BAL fluids indicated that a significant percentage of infiltrating leukocytes were eosinophils (Figure 4C, PC panel).

**Figure 5:**
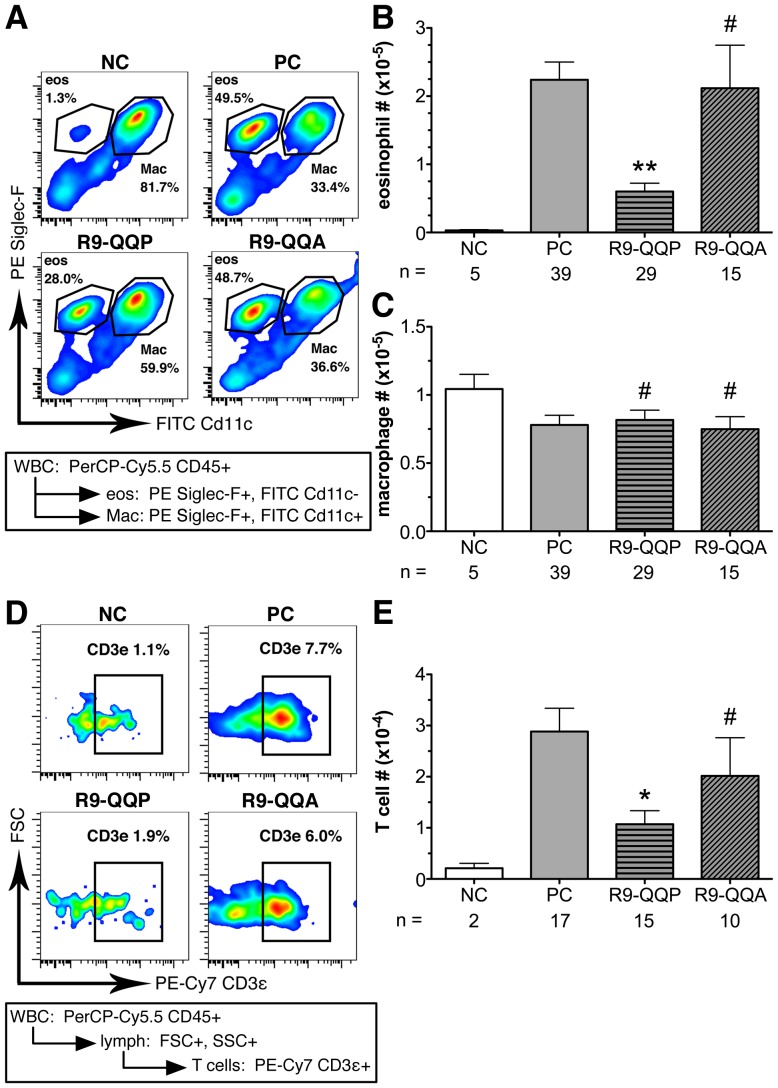
R9-QQP specifically inhibits eosinophilia. (A) Representative flow cytometric density plots obtained by specific antibody staining of BAL fluid cells from mice treated as indicated in Figure 2A and detailed in Materials and Methods. Inset box at bottom of graph outlines staining and analysis strategy: CD45^+^ cells gated and analyzed for Siglec-F and CD11c expression as described in Materials and Methods. Gates depict eosinophils and macrophages as percentages of total CD45^+^ cells. (B,C) Average (± SEM) number of eosinophils and macrophages respectively, calculated as described in Materials and Methods for the indicated number (n) of mice that had been treated as in panel (A). (D) Separate aliquots of BAL fluid cells from the experiment depicted in panel (A) analyzed as indicated in the inset box at the bottom of the graph: CD45^+^ cells gated by scatter and analyzed for CD3ε^+^ cells as described in Materials and Methods. Gates depict T lymphocytes as percentage of total CD45^+^ cells. (E) Average (± SEM) number of T cells calculated as described in Materials and Methods for the indicated number (n) of mice that had been treated as in panel (D). Axes in all histograms are displayed on a log scale for all fluorescence channels and on a linear scale for FSC. **, p < 0.0001; #, p>0.05; *, p<0.005.

In order to more precisely characterize as well as quantitate the cellular phenotype and frequency of infiltrating cells, respectively, we analyzed BAL fluid and lung cells by multi-color flow cytometry utilizing a staining approach previously reported by Stevens et al [14]. In these experiments, CD45-positive leukocytes that were stained for Siglec-F and CD11c allowed for the identification of eosinophils as defined by SiglecF-positive and CD11c-negative expression of these cell surface markers (Figure 5A). Asthmatic mice treated with R9-QQP displayed an almost two-fold decrease in the relative percentage of eosinophils found in BAL fluids (Figure 5A, compare PC and R9-QQP panels). For a more quantitative assessment of this decrease, we calculated the total numbers of eosinophils for multiple experiments using the indicated numbers of mice (Figure 5B). Compared to non-peptide treated animals (PC bar), R9-QQP treatment resulted in a 73% decrease in total eosinophils (Figure 5B). This significant reduction (p<0.05) was peptide specific, as it was not seen when mice were treated with the control peptide R9-QQA (Figure 5B). Furthermore, the same effect on eosinophilia was observed when cells isolated from lung tissue were analyzed with the same markers as above (data not shown). In contrast, the total numbers of alveolar macrophages were not affected upon R9-QQP treatment (Figure 5C). Using a similar multicolor FACS-based approach, the percentage of T cells was measured from BAL cells that were stained for expression of CD45 and the TCR/CD3ε (Figure 5D). The effect of R9-QQP treatment on infiltrating T cells mirrored that of eosinophils. That is, enumeration of total T cell numbers in BAL fluids showed a reduction of 63% upon R9-QQP treatment that was significant at p<0.05 (Figure 5E). This effect was specific for R9-QQP, as treatment with the control peptide R9-QQA had no significant effect on T cell infiltration (Figure 5E).

### R9-QQP treatment inhibits Th2 cytokine production by lymphoid cells from lung-draining lymph nodes

**Figure 6:**
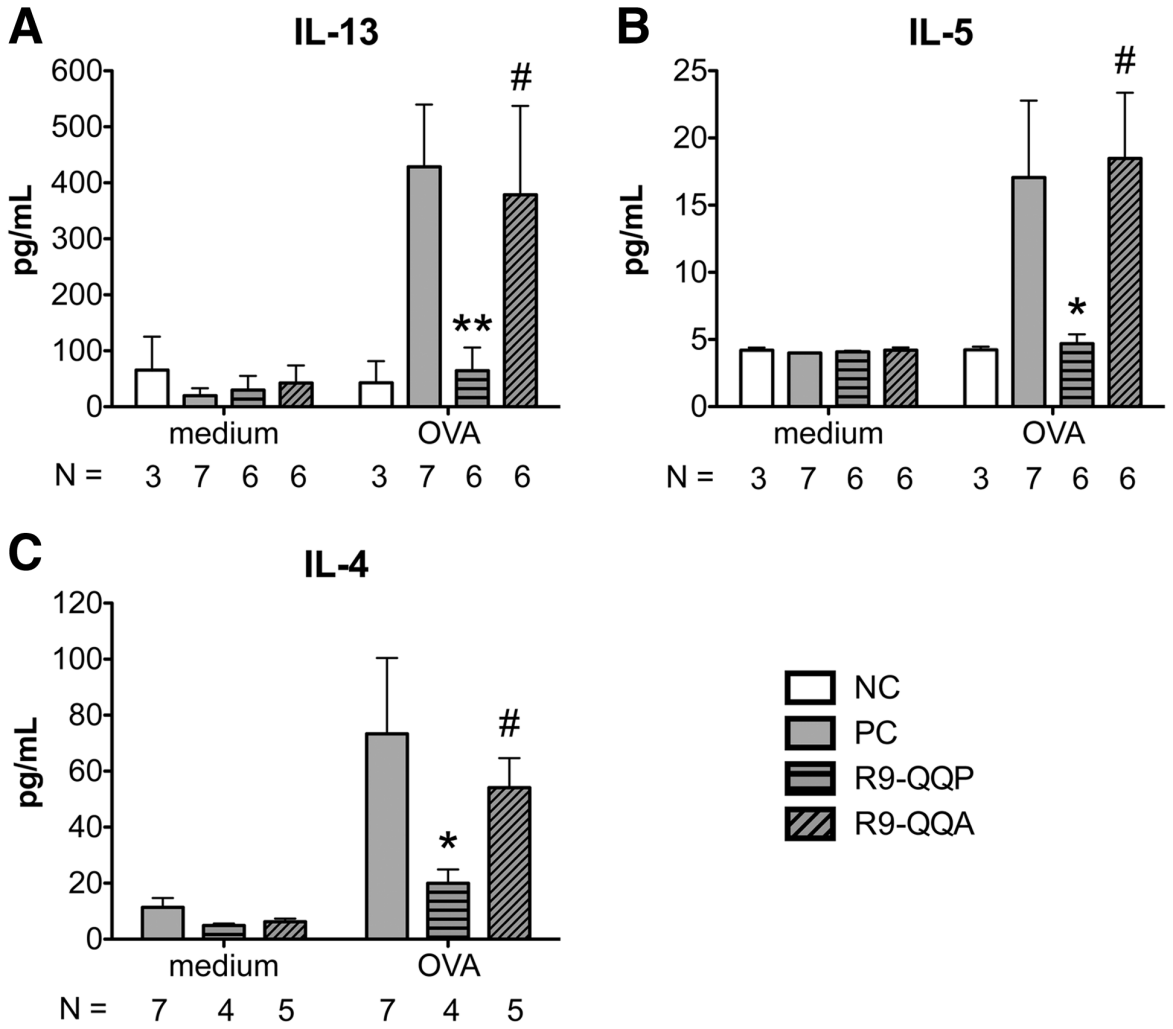
R9-QQP inhibits a lung DLN Th2 cytokine response. Cytokine levels of (A) IL-13, (B) IL-5, and (C) IL-4 in cultures of OVA (50 µg/ml) or medium restimulated lung-draining lymph node cells obtained from mice treated as described in Figure 2A and indicated in the inset legend. One×10^5^ (panels A and B) or 10^6^ (panel C) cells per well of round-bottom 96-well microtiter trays were incubated for 4 days at 37°C and cell-free culture supernatants were assayed by ELISA as described in Materials and Methods. Results are displayed as the average (± SEM) cytokine amounts for the indicated number (N) of independent experiments each representing DLN cells pooled from 2-4 mice. **, p < 0.01; #, p>0.05; *, p < 0.05.

T lymphocytes that are recruited to the lungs in response to OVA stimulation and challenge (Figure 5D and E) secrete Th2 cytokines such as IL-4, IL-5, and IL-13 (Figure 6). Among other events, sustained levels of these cytokines are known to induce eosinophilia, goblet cell hyperactivity and mucus production [16], [17]. Since ITK is known to regulate the transcriptional activation and secretion of Th2 cytokines [2], [3], we tested the effects of R9-QQP treatment on the ability of antigen-specific T cells from OVA stimulated and challenged mice to produce IL-4, IL-5 and IL-13. Due to the limited numbers of T cells recruited to the inflamed lung tissue, we isolated T cells from the lung draining lymph nodes (DLN). Isolated lymphocytes from the peribronchial and mediastinal DLN of OVA-sensitized and control mice that had been treated with R9-QQP, no peptide, or R9-QQA control peptide were re-stimulated *in vitro* with OVA, but without additional peptide treatment. The results in Figure 6 demonstrate that all three Th2 cytokines were significantly reduced in cultures prepared from the DLN of mice that were treated with R9-QQP but not those from the DLN of mice that were treated with control peptide or no treatment. Reduction was 85% in the case of IL-13 (Figure 6A), 71% for IL-5 (Figure 6B) and 73% for IL-4 (Figure 6C).

## Discussion

The Inducible T cell kinase (ITK) is a major regulator of Th2 cytokine production and mice deficient in ITK (ITK-KO) fail to express Th2 effector function upon activation in the absence of cytokine receptor-mediated signaling [2], [4], [18]. The TCR-induced activation of ITK requires the interaction of the kinase with the adaptor protein SLP-76 [11], [10], [13]. Previous studies utilizing *in vitro* experimental systems or mice expressing mutated genes of SLP-76 have shown that the TCR-induced association between ITK and SLP-76 is co-operative, involving specific interactions between the ITK-SH2 domain and pTyr145 on SLP-76, as well as the interaction between the ITK-SH3 domain and its polyproline ligand on SLP-76 [9], [10], [13]. Jordan et al, utilizing mice bearing a mutation on tyrosine 145 (Y145F) of SLP-76, found that even though the TCR-induced association of ITK with SLP-76 was not perturbed, the activation of ITK was significantly compromised [10]. In our previous *ex vivo* studies, we demonstrated that the synthetic peptide R9-QQP, a specific competitive inhibitor of the ITK-SH3/SLP-76 poly-proline interaction, disrupted both the association between these two signaling partners, as well as the activation of ITK and subsequent Th2 cytokine production [12]. In these studies the R9-QQP mediated inhibition was specific for ITK, as other signaling partners such as LCK and ZAP-70 were not affected. These observations are consistent with the hypothesis that the interaction between ITK-SH3 and the poly-proline region of SLP-76 is a prerequisite for the interaction of the ITK-SH2 domain and Tyr-145 of SLP-76 and for the trans-phosphorylation and activation of ITK [12].

To further substantiate the above hypothesis, we have assessed the significance of the interaction between the SH3 domain of ITK and the poly-proline region of SLP-76 during an *in vivo* immune response as represented by lung inflammation in a murine model of allergic asthma. We found that treatment of mice with R9-QQP causes significant inhibition of the inflammatory immune response as manifested by airway hyper-responsiveness, inflammatory cell infiltration, bronchial mucus accumulation, and production of relevant cytokines from draining lymph nodes. The observed inhibition of these parameters is R9-QQP peptide specific because cell-permeable control peptides with scrambled amino acid sequence (R9-PQM) or peptides with substitutions of the critical proline residues with alanine (R9-QQA) lacked any significant effect. As it has been previously demonstrated, R9-QQP targets and disrupts the interaction between the SH3 domain of ITK and the poly-proline region of SLP-76 [12]. Although SLP-76 can interact with other SH3 domain-containing intracellular partners, R9-QQP specifically interferes with the interaction of the ITK-SH3 domain and the poly-proline region of SLP-76, as evidenced by the fact that the association between SLP-76 and LCK, and SLP-76 and GADS are not affected by R9-QQP [12]. The possibility that R9-QQP may not specifically affect T cells, but also other immune cells that may express ITK is very unlikely because there is no evidence that the interaction between ITK and SLP-76 occurs in cells other than T lymphocytes.

Accumulation of eosinophils into the lungs represents a hallmark sign of lung inflammation in allergic asthma [19]. The observation that inhibition of ITK activation by R9-QQP causes significant reduction in OVA-inducible eosinophilia (Figure 5A and B) further substantiates the critical role that ITK plays in an immune response where Th2 cytokines are involved such as the allergic inflammatory response. Unlike eosinophils, no significant effect was noted on the number of lung-resident alveolar macrophages (Figure 5C). Thus, the apparent reduction in the relative percentage of macrophages (Figure 5A) is most likely due to the large influx of inflammatory cells into the lungs of the allergic animals. It is known that the recruitment of eosinophils into local sites of inflammation involves several cytokines, most notably the Th2 cytokines IL-4, IL-5, and IL-13 that are produced by CD4^+^ T lymphocytes [20]. Therefore, it was not surprising to find that R9-QQP treatment significantly reduced recruitment of T cells to the lungs of allergic mice (Figure 5D and E).

Previous observations have suggested that ITK deficiency results in the inability to produce Th2 cytokines in antigen recall responses [2], [21]. Consistent with this known role of ITK, it is interesting to note that a Th2 cytokine recall response by splenocytes from OVA-immune mice was significantly reduced upon treatment with R9-QQP *in vitro* (Figure 1). Furthermore, re-stimulation of lymphoid cells from lung draining lymph nodes of R9-QQP-treated, OVA-asthmatic mice also displayed reduced Th2 cytokine responses upon *in vitro* re-stimulation (Figure 6). Thus, similar to the situation in which ITK is genetically ablated (ITK-KO), blocking the TCR-inducible activation of ITK by means of treatment with the competitive inhibitory peptide R9-QQP results in a similar reduction of Th2 recall responses.

The current study targets the ITK/SLP-76 interaction in the context of an *in vivo* immune response, as manifested during allergic inflammation, and identifies it as an important amplification step of signal transduction downstream of the TCR. In contrast to genetic ablation (ITK-KO), we studied the role of ITK in the initiation of an *in vivo* immune response using mice in which T-cell development and migration of CD4+ lymphocytes into peripheral circulation was allowed to occur unperturbed. Several interpretations may in part or in concert account for the reduced lung inflammation reported in the current work. As R9-QQP in the current study was delivered to mice at the stages of sensitization and challenge to OVA, possible interpretations include a necessary role for ITK in the proliferation of OVA-specific CD4+ T cells clones during sensitization. Separately, our results include the possibility that migration of Th2 effectors to the local site of inflammation in the lung tissue is affected upon *in vivo* inhibition of ITK; this possibility is consistent with a previously described role of ITK in TCR-dependent actin polymerization as well as chemokine-receptor mediated signaling [22]–[24]. Finally, ITK is likely required *in vivo* for the sustained release of pathogenic Th2 cytokines upon re-stimulation with OVA during intranasal exposure at the challenge period. Evidence to support this last model is provided by results presented here demonstrating R9-QQP-specific inhibition of Th2 cytokines upon *ex vivo* treatment of splenocytes from OVA-sensitized mice followed by re-stimulation and cytokine secretion occurring *in vitro* (Figure 1). Future studies in our laboratory aim to test these separate models in order to further refine the requirement of ITK in asthma pathogenesis.

Increased levels of IgE during allergic lung inflammation have been shown to play a role in human disease [25]. In additional experiments (data not shown), we observed that OVA sensitization and challenge induced an increase in the levels of total serum IgE, however, treatment with R9-QQP had no effect on IgE levels unlike the reduction observed for parameters of lung inflammation. One interpretation that could account for these results could be that inhibition of ITK by R9-QQP may result in sufficient reduction of sustained levels of Th2 cytokines to alleviate the symptoms at the local site of inflammation occurring in the lung tissue, but not sufficient to inhibit T cell-dependent isotype switching to IgE occurring in the B cell germinal center microenvironment of the lung draining lymph nodes. This interpretation is consistent with the data of Mueller and August who used a similar animal model to ours and observed that levels of OVA-specific serum IgE obtained from ITK-deficient mice were not significantly affected as compared to WT mice despite of reduced lung inflammation in the ITK-KO mice [5]. Finally, this interpretation is also consistent with other data in which increased IgE levels and mast cells did not always accompany lung inflammation in animal models such as the one utilized in the current study [26].

Other investigators have previously used chimeric peptides containing poly-arginine cationic domains for the intracellular delivery of inhibitory cargos. A notable example is the inhibition of the inducible nuclear translocation of NFAT, its transcriptional activity, and IL-2 production through the delivery of a specific peptide able to inhibit the interaction between calcineurin and NFAT [27]. This inhibitory peptide was conjugated to eleven arginine residues and, upon intraperitoneal injection, was able to prolong mismatched pancreatic islet allografts in mice in a transplant rejection model [27]. Another example is the inhibition of phosphorylation of cAMP responsive element-binding protein and long-term neuronal potentiation by a PKA inhibitory peptide that was delivered to the nuclei of neurons by a fusion peptide containing eleven arginines [28]. In addition, topical application of a conjugate of heptameric arginine and cyclosporine A successfully treated cutaneous inflammation in a murine model of contact dermatitis [29].

In conclusion, the present findings are significant in that 1) they provide the first demonstration of the biological importance of the SH3 domain-mediated interaction between ITK and SLP-76 in the activation of ITK and the subsequent Th2 cytokine production in a disease model, 2) they underscore the potential of cell permeable peptides as useful research tools for investigating signaling pathways in disease models, and 3) they provide translational implications for targeting ITK in the design of potential novel molecular therapies.
